# Supplementary material for: Endothelial discoidin domain receptor 1 senses flow to modulate YAP activation
Source: Nat Commun. 2023 Oct 13;14:6457. doi: 10.1038/s41467-023-42341-z (PMC10576099; doi:10.1038/s41467-023-42341-z)
Supplement: Supplementary file 1 — Supplementary Information [file 41467_2023_42341_MOESM1_ESM.pdf]

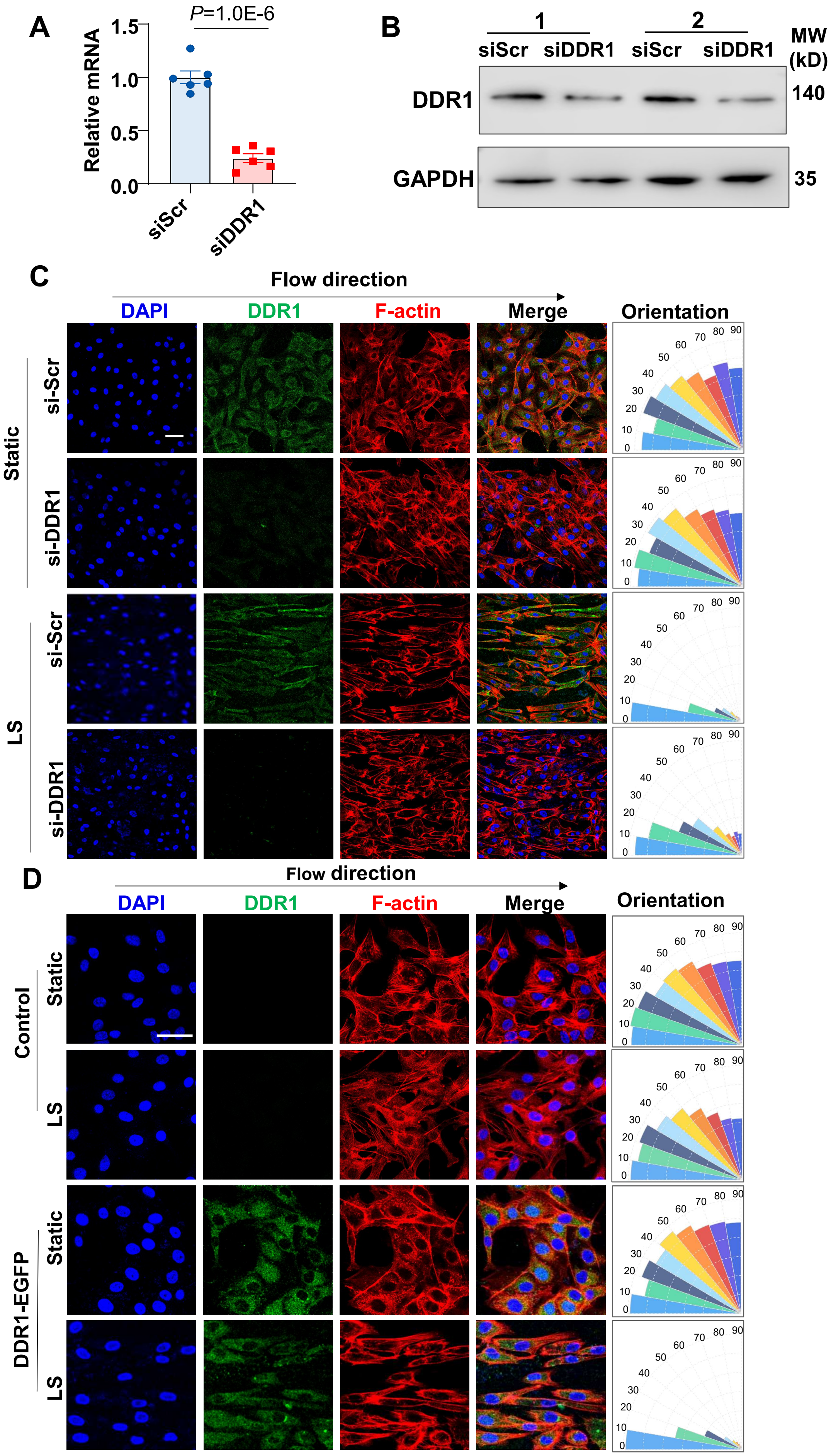


**Figure S1. DDR1 is required for EC alignment with flow.**

**Figure S1. DDR1 is required for EC alignment with flow.**
(A) The knockdown efficiency of DDR1 in HUVECs assessed by RT-qPCR. n=6 biological replicates. Data were expressed as the means ± SEM and analyzed by two-tailed unpaired t-test. (B)The knockdown efficiency of DDR1 confirmed by western blotting in HUVECs transfected with siRNAs specific for DDR1 or scrambled siRNA. Two biological replicates were showed. (C) Left: Immunofluorescence of F-actin and DDR1 in HUVECs transfected with DDR1-siRNA or scrambled control siRNA and subjected to static condition or laminar shear for 24h. Right: Quantification of alignment by measuring the orientation angle. n = 9 images from 3 biological replicates. (D) Left: Immunofluorescence of F-actin and DDR1 in NIH3T3 cells infected with DDR1-EGFP adenovirus and subjected to static condition or laminar shear for 24h. Right: Quantification of alignment by measuring the orientation angle. n = 9 images from 3 biological replicates. Scale bars, 50 μm.

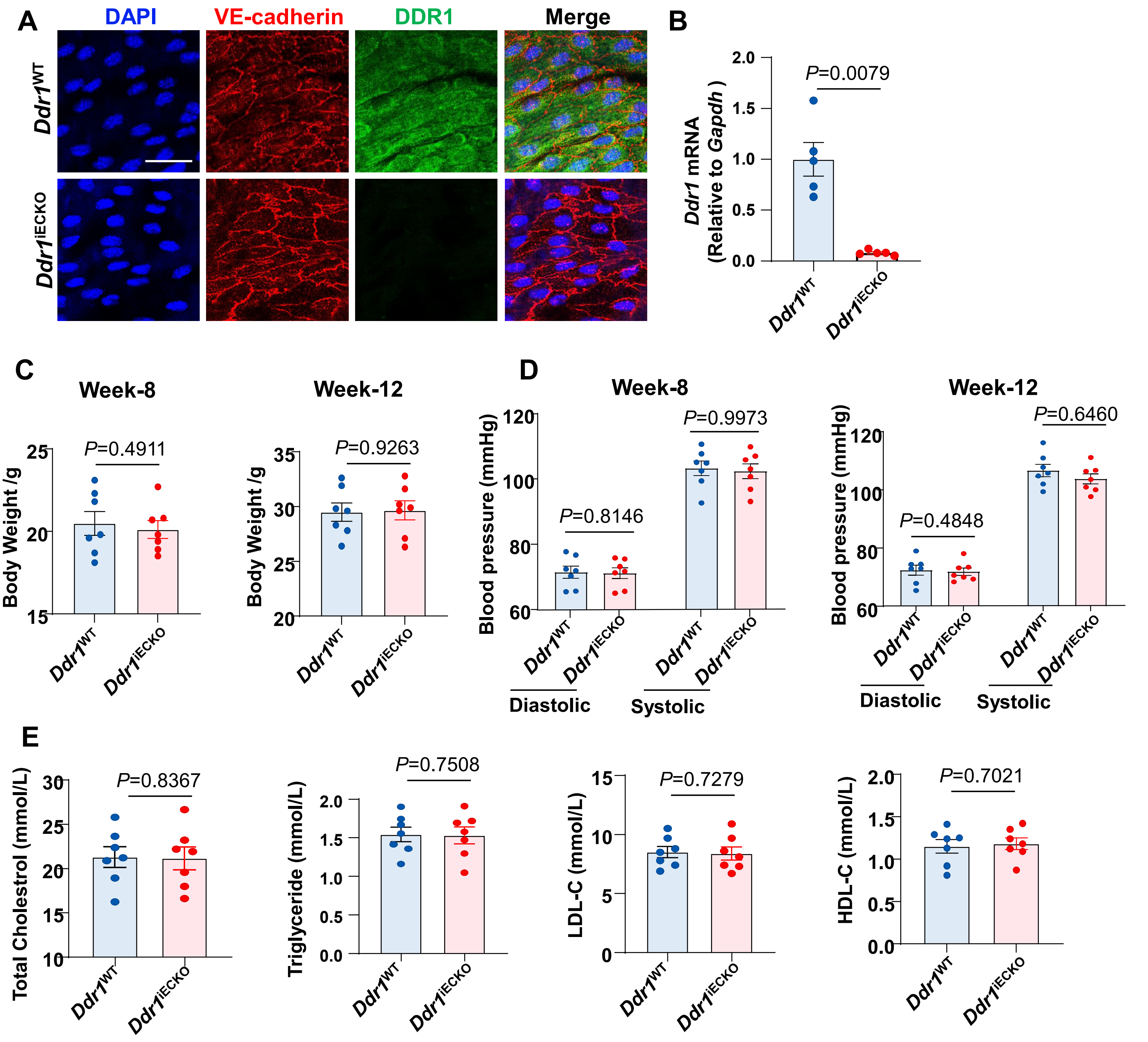


**Figure S2. Bodyweights, blood pressures and lipid profile analysis of *Ddr1*$^{WT}$ and *Ddr1*$^{iECKO}$ mice.**

(A) En-face staining to determine the expression of DDR1 in the aortas of *Ddr1*$^{WT}$ and *Ddr1*$^{iECKO}$ mice at 1 week after tamoxifen injection. Scale bar, 50 μm. 3 biological replicates were conducted with similar results. (B) RT-qPCR to verify the efficiency of Ddr1 depletion in *Ddr1*$^{iECKO}$ mice compared with *Ddr1*$^{WT}$ mice at 1 week after tamoxifen injection. n=5 mice. Data were expressed as the means ± SEM and analyzed by Mann-Whitney test. (C) Body weight of *Ddr1*$^{WT}$ and *Ddr1*$^{iECKO}$ mice at week-8 (before surgery) and week-12 (4-wk postsurgery). (D) Systolic and diastolic blood pressures of *Ddr1*$^{WT}$ and *Ddr1*$^{iECKO}$ mice at week-8 and week-12. (E) Total serum cholesterol, triglyceride, LDL-C, and HDL-C level in *Ddr1*$^{WT}$ and *Ddr1*$^{iECKO}$ mice at 4-wk postsurgery. In (C-E), Data were expressed as the means ± SEM and analyzed by unpaired t-test, n=7 mice.

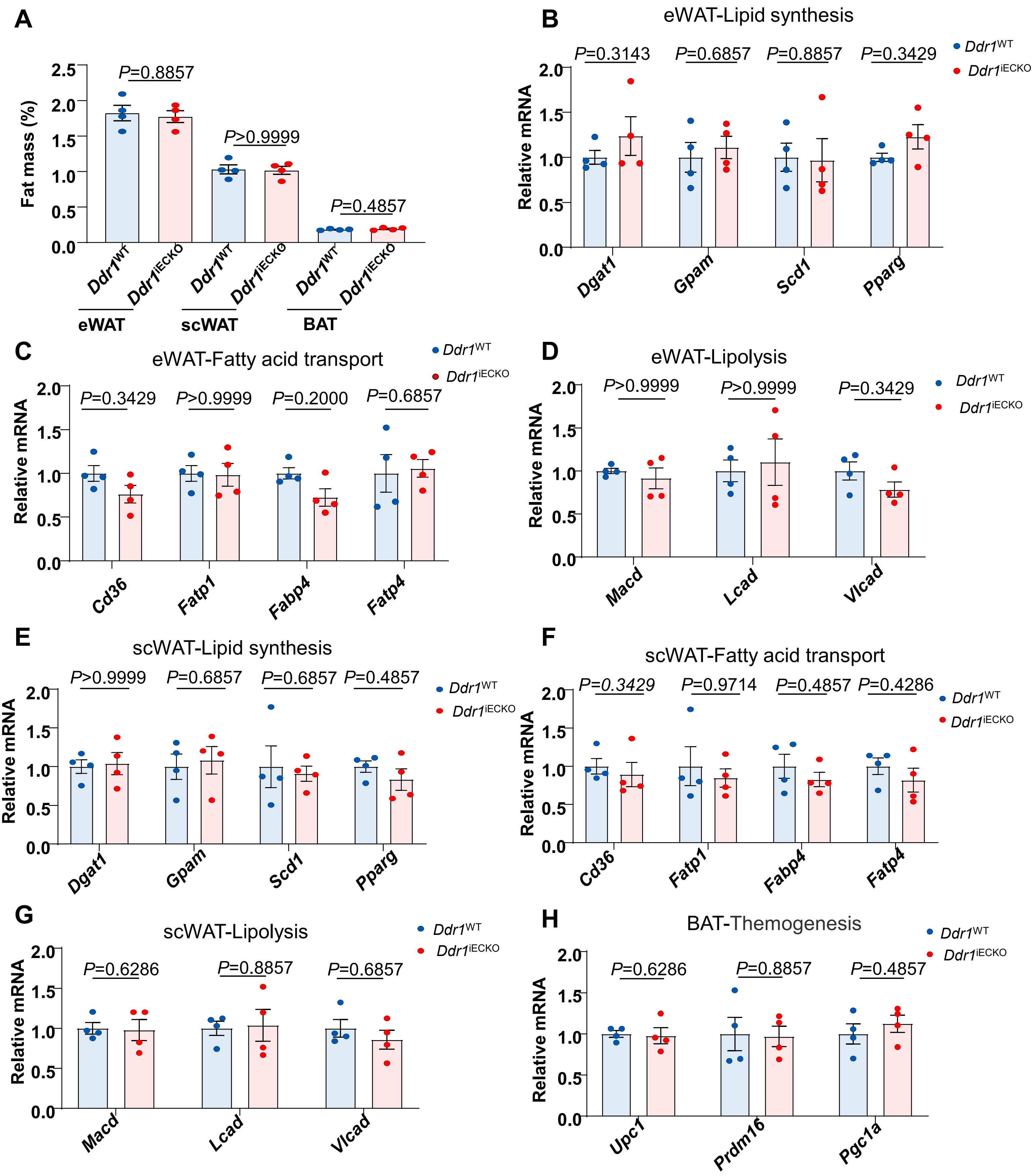


**Figure S3. Cdh5-specific DDR1 knockout does not exert a discernible influence on lipid metabolism.**

(A) The weight-to-body weight ratio of epididymal white adipose tissue (eWAT), subcutaneous white adipose tissue (scWAT) and brown adipose tissue (BAT) in western-diet-fed *Ddr1* $^{WT}$ and *Ddr1* $^{iECKO}$ mice. (B-D) RT-qPCR to detect lipid synthesis (B), fatty acid transport (C) and lipolysis (D) of eWAT. n=4 mice. (E-G) RT-qPCR to detect lipid synthesis (E), fatty acid transport (F) and lipolysis (G) of scWAT. n=4 mice. (H) RT-qPCR to detect the themogenesis of BAT. n=4 mice. Data were all expressed as the means ± SEM and analyzed by Mann-Whitney test.

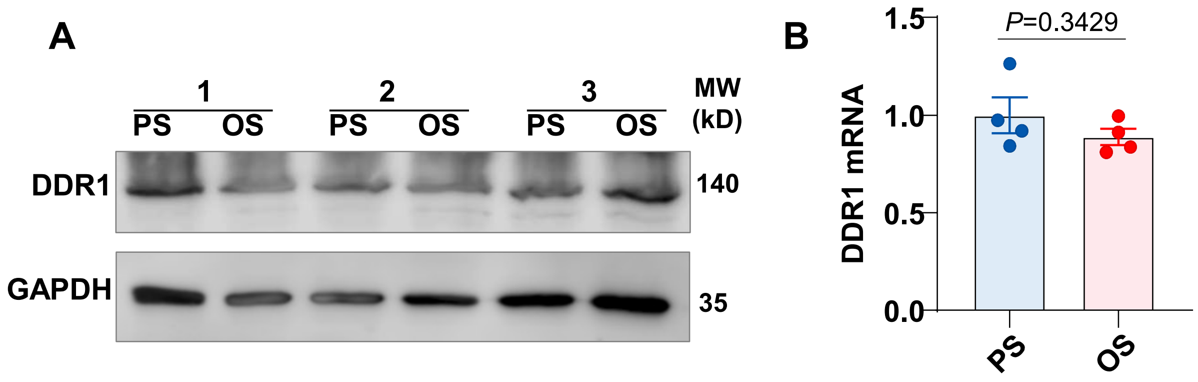


**Figure S4. Shear stress does not affect the expression of DDR1 in endothelial cells.** (A) Western blotting to detect DDR1 expression in HUVECs subjected to PS or OS for 24h. 3 biological replicates were presented. (B) Quantitative RT-PCR analysis of the expression of DDR1 in HUVECs treated as in (A). n=4 biological replicates. Data were expressed as the means ± SEM and analyzed by Mann-Whitney test.

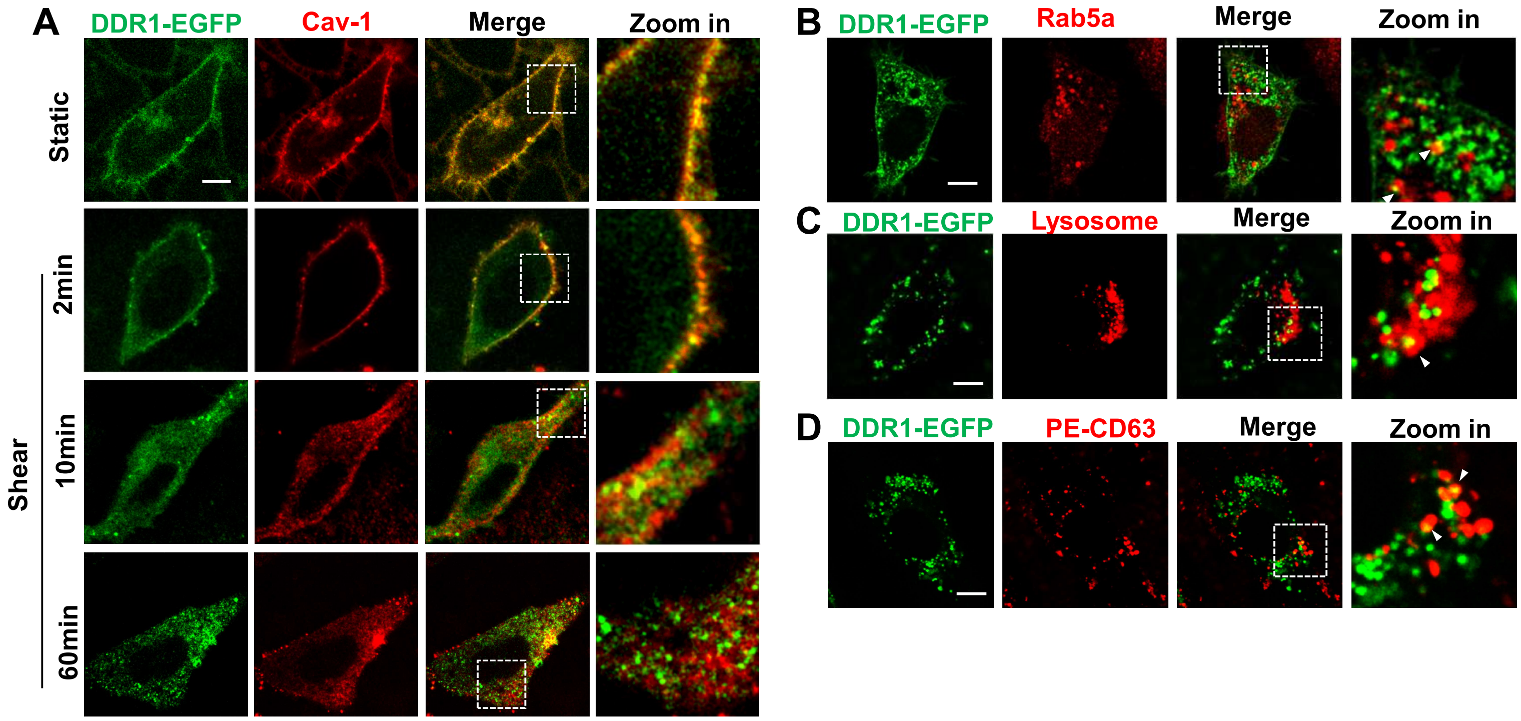


**Figure S5. The relationship between DDR1 droplets and lipid rafts, endosomal or exocytotic vesicles.**

(A) Immunofluorescence of DDR1-EGFP and lipid raft marker caveolin-1 (Cav-1) in HUVECs, which were infected with DDR1-EGFP adenovirus and subjected to laminar shear. (B) Immunofluorescence of DDR1-EGFP and early endosome marker Rab5A in HUVECs, which were infected with DDR1-EGFP adenovirus and subjected to laminar shear for 1h. (C) Immunofluorescence of DDR1-EGFP and Lyso-Tracker Red in HUVECs, which subjected to laminar shear for 1h. (D) Immunofluorescence of DDR1-EGFP and exosomes marker CD63 in HUVECs, which subjected to laminar shear for 1h. Scale bars, 10 μm. In A-D, experiments were repeated 3 times independently with similar results.

.

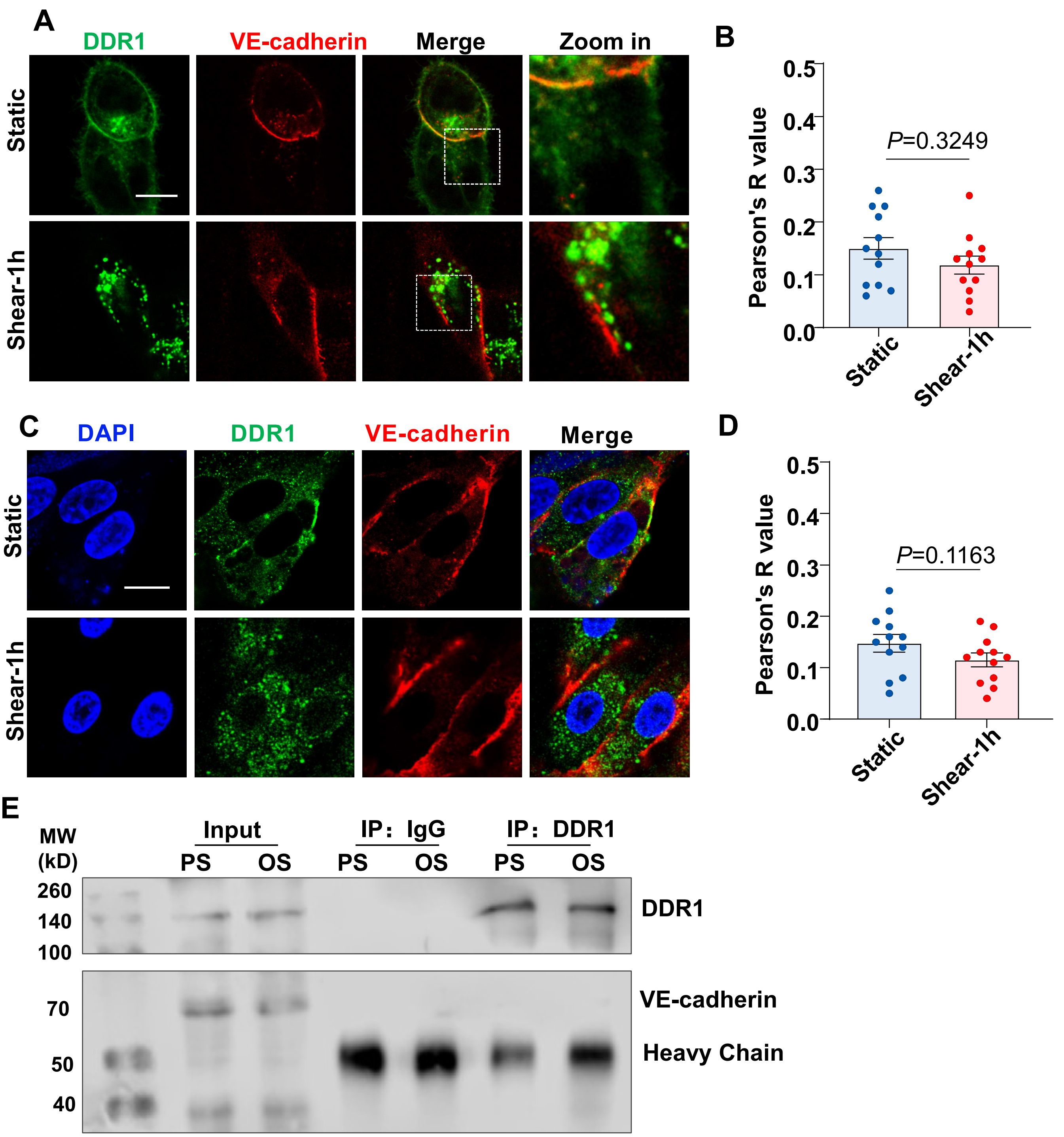


**Figure S6. DDR1 has no interaction with VE-cadherin in endothelial cells.**
(A) Live cell imaging of DDR1-EGFP and VE-cadherin-Cherry in ECs, which were grown in microfluidic chamber and subjected to laminar shear stress. (B) Colocalization analysis of DDR1-EGFP and VE-cadherin-Cherry in (A). Pearson's R value (above threshold) was calculated by ImageJ Fiji software (Analyze-colocalization-coloc2). n = 12 images from 3 biological replicates. (C) Immunofluorescence staining of DDR1 and VE-cadherin in HUVECs subjected to laminar shear stress for 1h or static. (D) Colocalization analysis of DDR1 and VE-cadherin in (C). n = 12 images from 3 biological replicates. Data were all expressed as the means ± SEM and analyzed by Mann-Whitney test. (E) Co-IP assay to detect the interaction between DDR1 and VE-cadherin in HUVECs subjected to PS or OS for 24h. Scale bars, 20 μm.

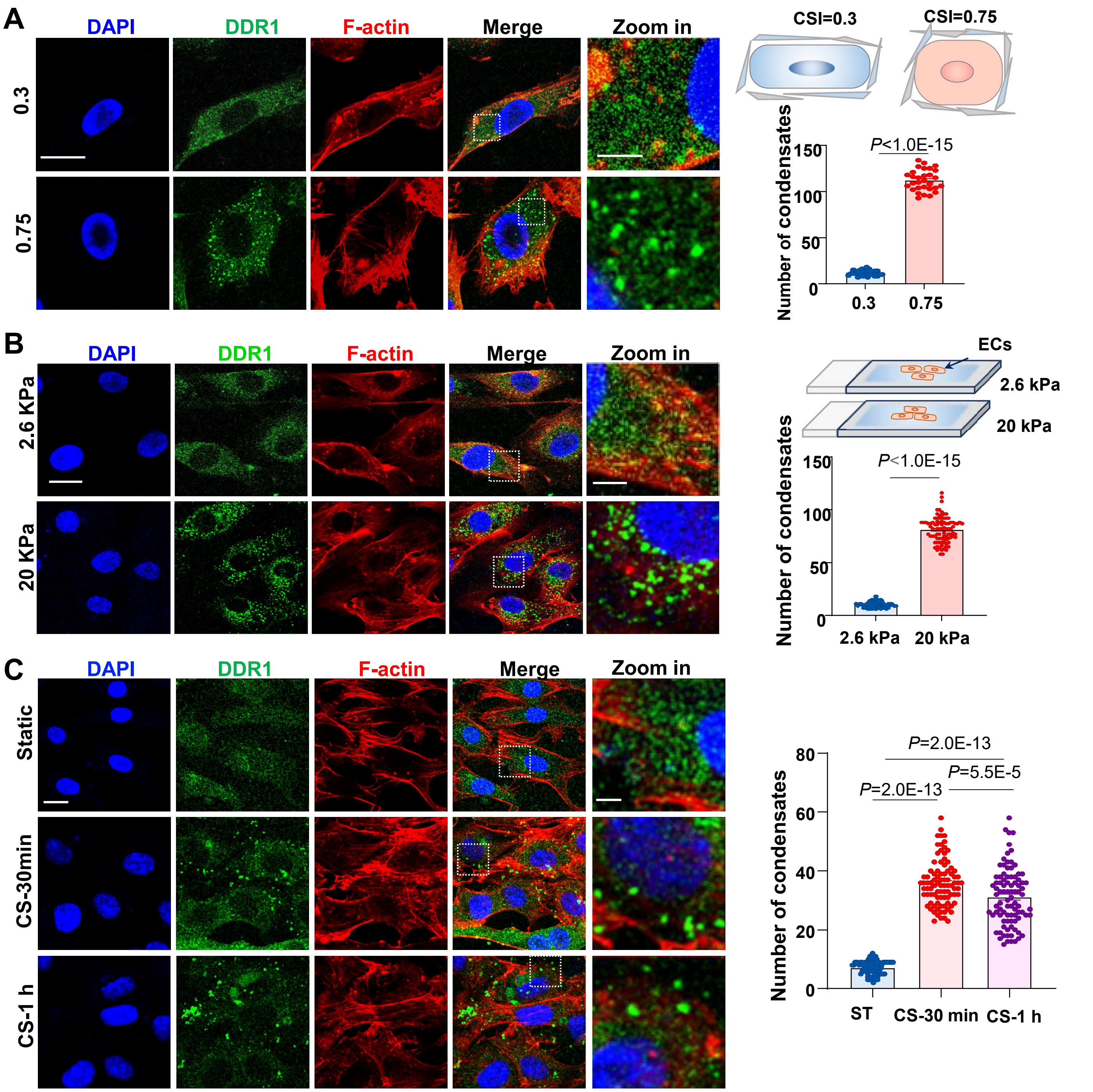


**Figure S7. DDR1 can respond to various mechanical stimuli in endothelial cells.**
(A) Immunofluorescence and quantification of DDR1 condensates in micropatterned (CSI = 0.30 or CSI = 0.75) HUVECs, PDMS stamp with micropatterned was coated with gelatin. CSI: cell shape index. n=30 cells from 3 biological replicates. Data were analyzed by unpaired t-test. (B) Immunofluorescence and quantification of DDR1 condensates in HUVECs seeded on gelatin-coated gels (2.6 and 20 kPa) for 24h. n=90 cells from 6 biological replicates. Data were analyzed by unpaired t-test. (C) Immunofluorescence and quantification of DDR1 condensates in HUVECs subjected to uniaxial cyclic stretch (15% deformation) or under the static condition. n=90 cells from 3 biological replicates. Data were analyzed by one-way ANOVA followed by Tukey's multiple comparisons test. Scale bars (overview), 20 μm. Data were all expressed as the means ± SEM. Scale bars (zoom in), 4 μm.

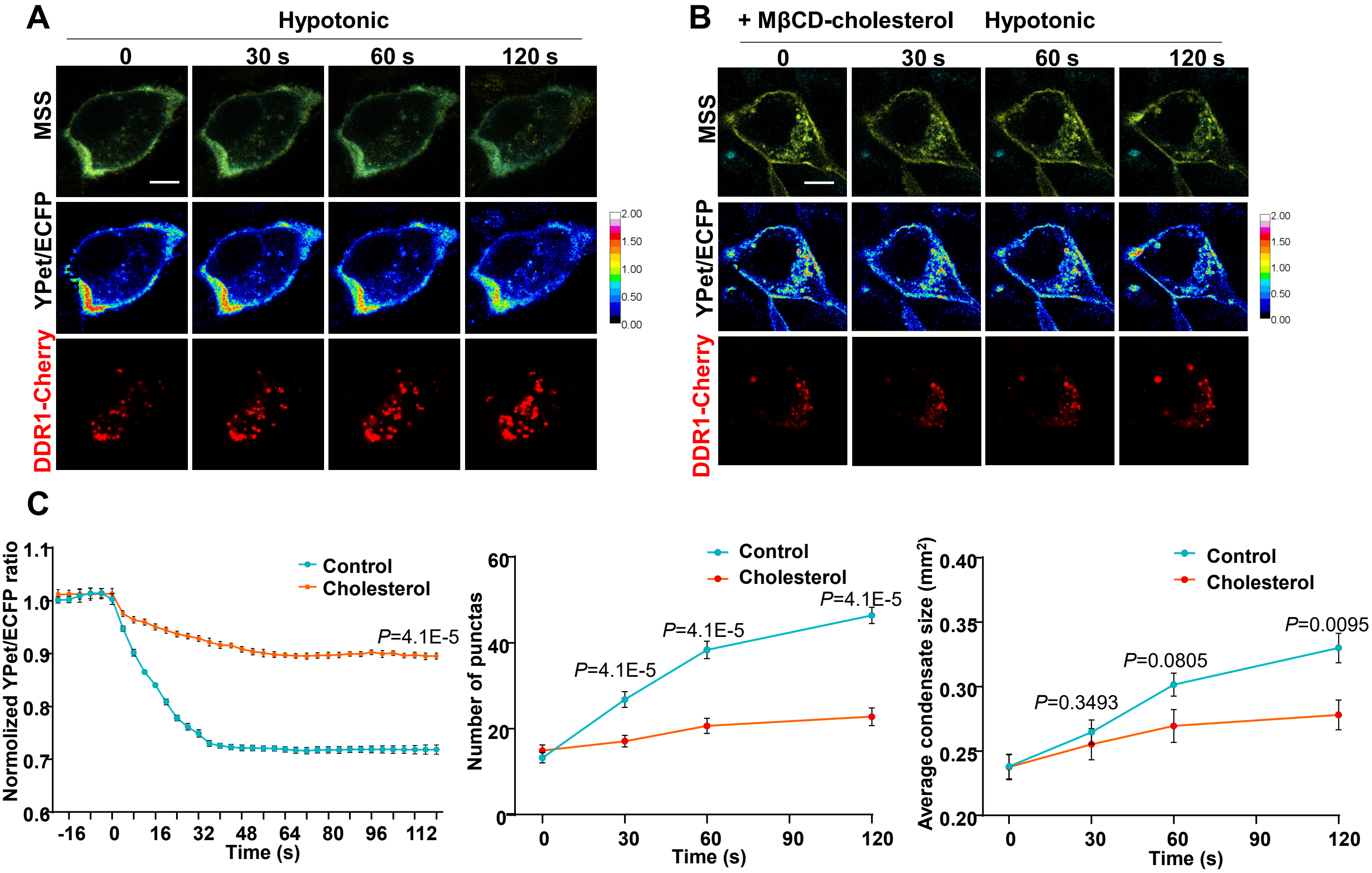


**Figure S8. Elevate membrane tension by hypotonic stimulus can promote the DDR1 droplet formation.**

(A) The representative living cell images of YPet/ECFP emission ratio and DDR1-Cherry in EA.hy926 ECs after exposure to hypotonic solution. (B) The representative living cell images of YPet/ECFP emission ratio and DDR1-Cherry in ECs pretreated with MβCD-cholesterol and then exposed to hypotonic solution. (C) The average time courses of FRET biosensors and quantification of number and size of DDR1 condensates in ECs exposed to hypotonic solution. n= 9 biological replicates. Data were expressed as the means ± SD, and analyzed by two-tailed Mann-Whitney test. Scale bars, 10 μm.

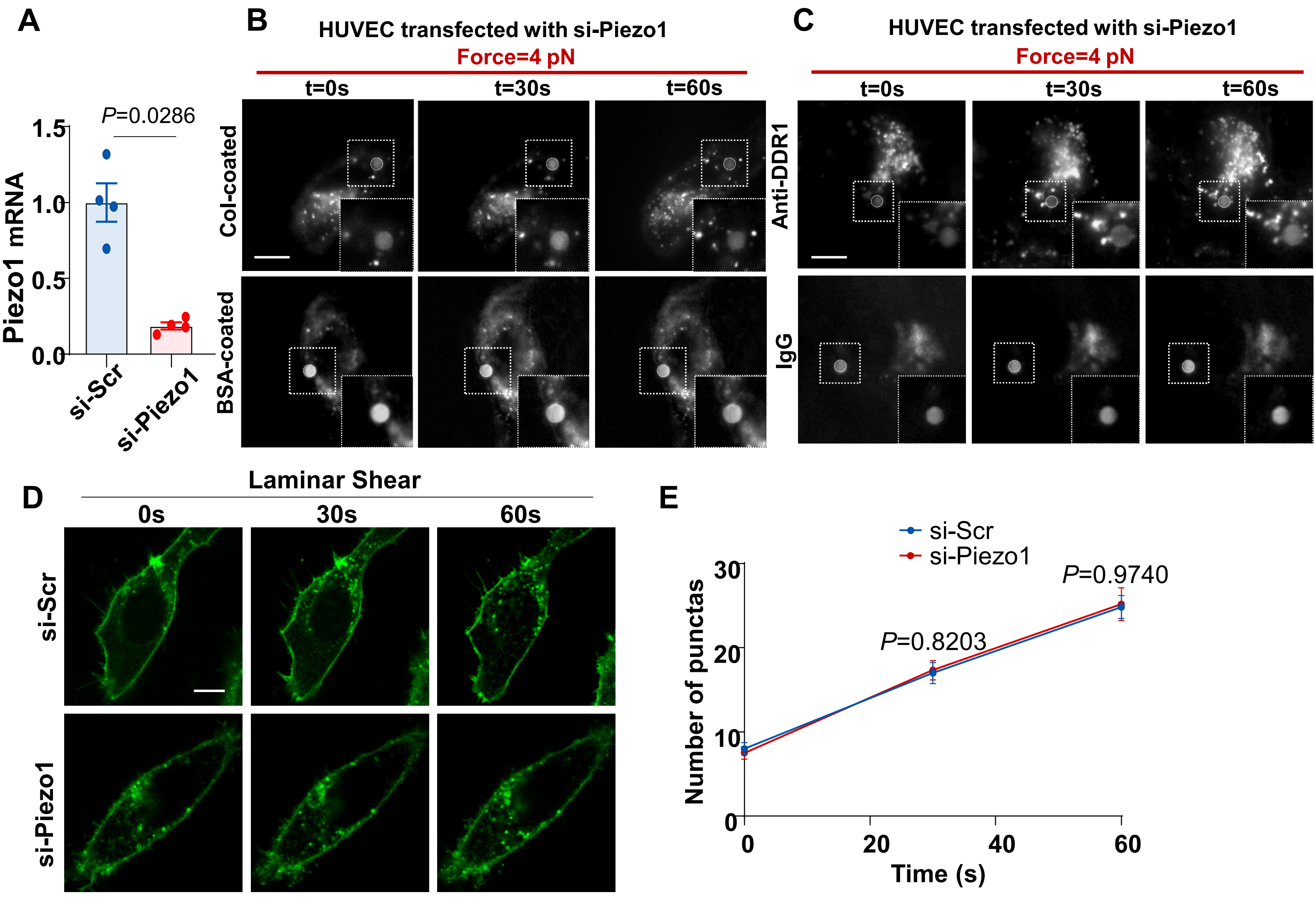


**Figure S9. Knocking down piezo1 does not affect the activation of DDR1.**
(A) The knockdown efficiency of DDR1 in HUVECs assessed by RT-qPCR. n=4 biological replicates. Data were expressed as the means ± SEM and analyzed by Mann-Whitney test. (B and C) Images of DDR1 condensates formation captured by total internal reflection fluorescence (TIRF) microscopy. HUVECs were transfected with Piezo1-specific siRNA and infected with DDR1 adenovirus. After 48 hours, cells were incubated with collagen/ BSA-coated beads (B) or anti-DDR1/IgG-coated beads (C). Scale bar, 5 μm. Experiments were repeated 3 times independently with similar results. (D) Time-lapse images of DDR1 in HUVECs infected with DDR1-EGFP adenovirus. Cell grown in gelatin-coated microfluidic chamber were subjected to laminar shear stress. (E) Quantification of DDR1 condensates number. Data were expressed as the means ± SEM. n=6 biological replicates. Data were analyzed by Mann-Whitney test. Scale bars, 10 μm.

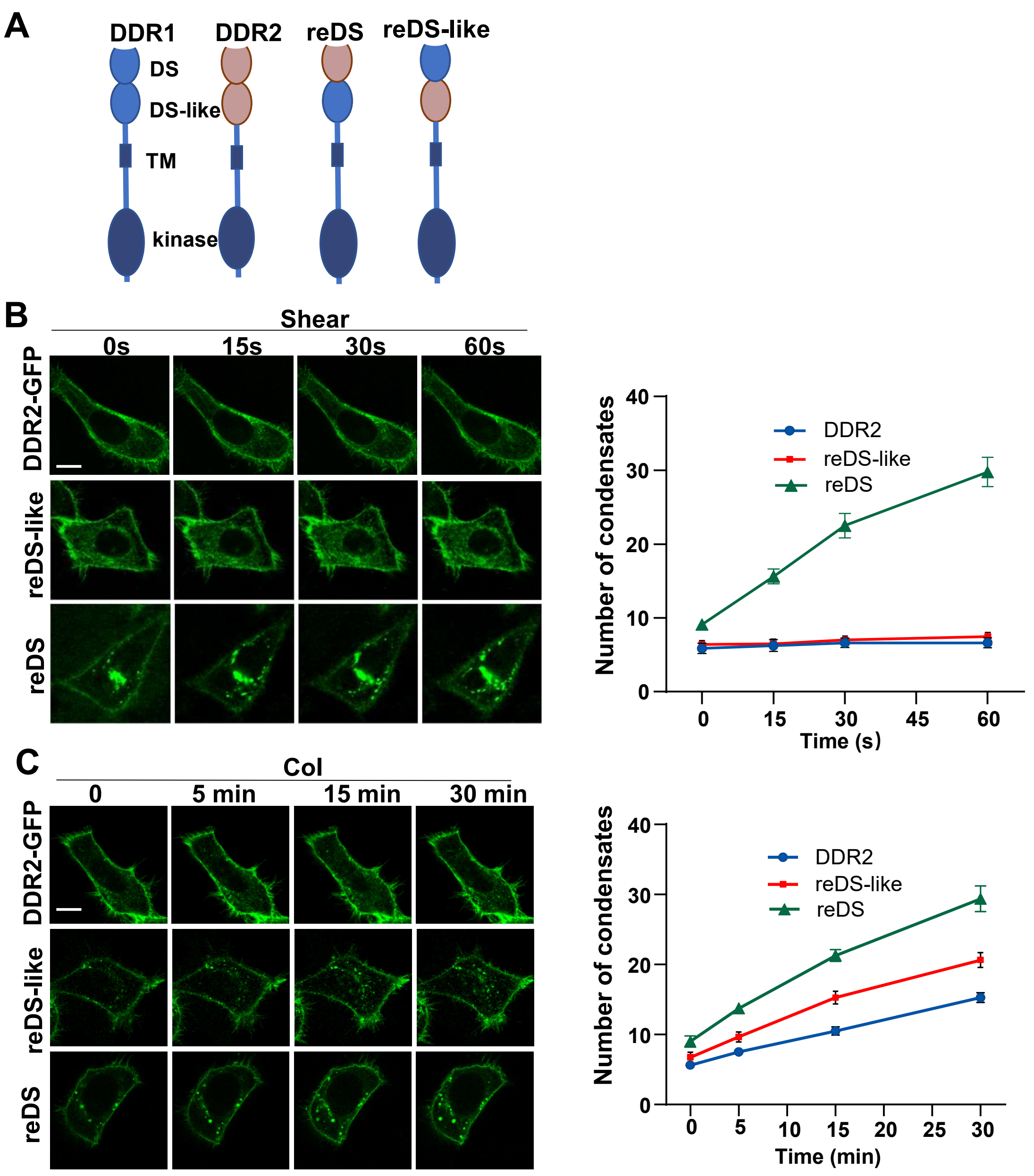


**Figure S10. DS-like domain of DDR1 is crucial for shear stress-induced DDR1 droplets formation.**

(A) Schematic diagram of DDR1, DDR2, reDS (the DS domain of DDR1 replaced by that of DDR2), reDS-like (the DS-like domain of DDR1 replaced by that of DDR2). (B) Left: live cell imaging of DDR2, reDS or reDS-like in EA.hy926 ECs exposed to laminar shear stress. Right: quantification of DDR1 condensates. n=8 biological replicates. Data were expressed as the means ± SEM. (C) Left: live cell imaging of DDR2, reDS or reDS-like in EA.hy926 ECs after 10μg/ml soluble collagen I stimulation. Right: quantification of DDR1 condensates. n=8 biological replicates. Data were expressed as the means±SEM. Scale bars, 10 μm.

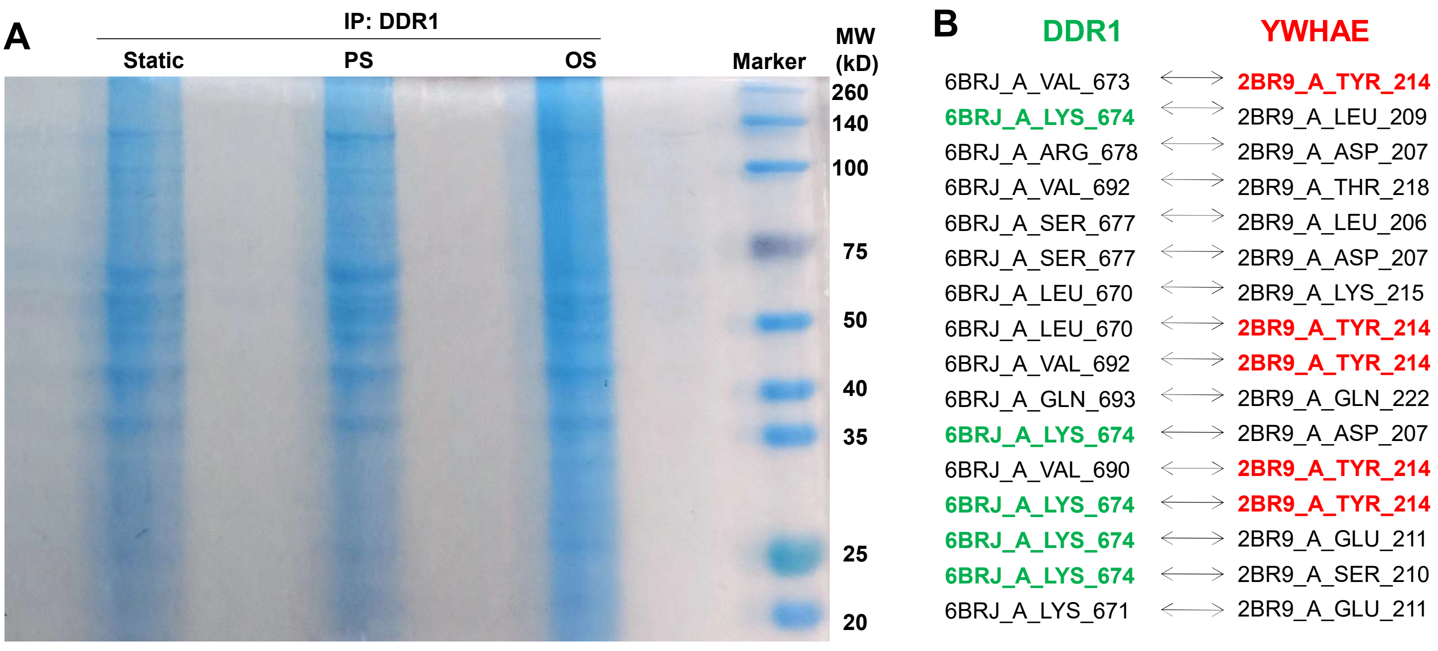


**Figure S11. DDR1 can interact with YWHAE by C-terminal domain.**
(A) Cellular extracts from HUVECs cultured under the static condition or subjected to PS or OS for 3h were subsequently immunoprecipitated with anti-DDR1 antibody. Eluted proteins were separated on SDS-PAGE and visualized by Coomassie blue staining. Eluted proteins were identified by mass spectrometry analysis. (B) Interface residues contacts of 6BRJ (605~913aa of DDR1) and 2BR9 (1~233aa of YWHAE) predicted by PRISM. The residues Lys674 of DDR1 and Tyr214 of YWHAE were highlighted in green and red, respectively.

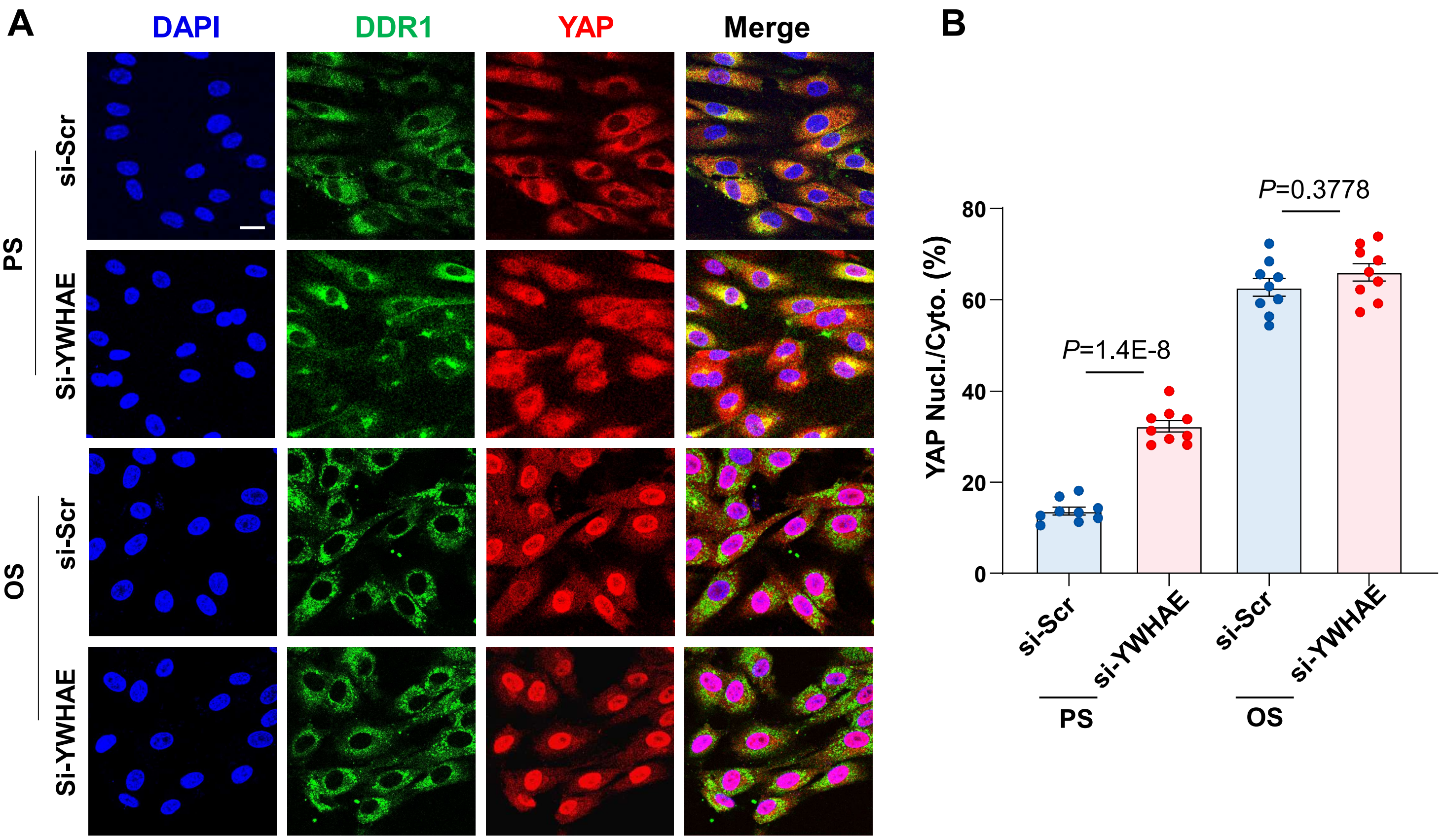


**Figure S12. The elimination of YWAHE promotes the nuclear translocation of YAP.**
(A) Representative Immunofluorescence staining of DDR1 and YAP1 in HUVECs subjected to PS or OS for 24 hours. (B) Quantification of YAP localization in (A). n= 9 images from 3 biological replicates. 10~15 cells per image. Data were expressed as the means ± SEM and analyzed by two-way ANOVA followed by Tukey's multiply test. Scale bar, 20 μm.

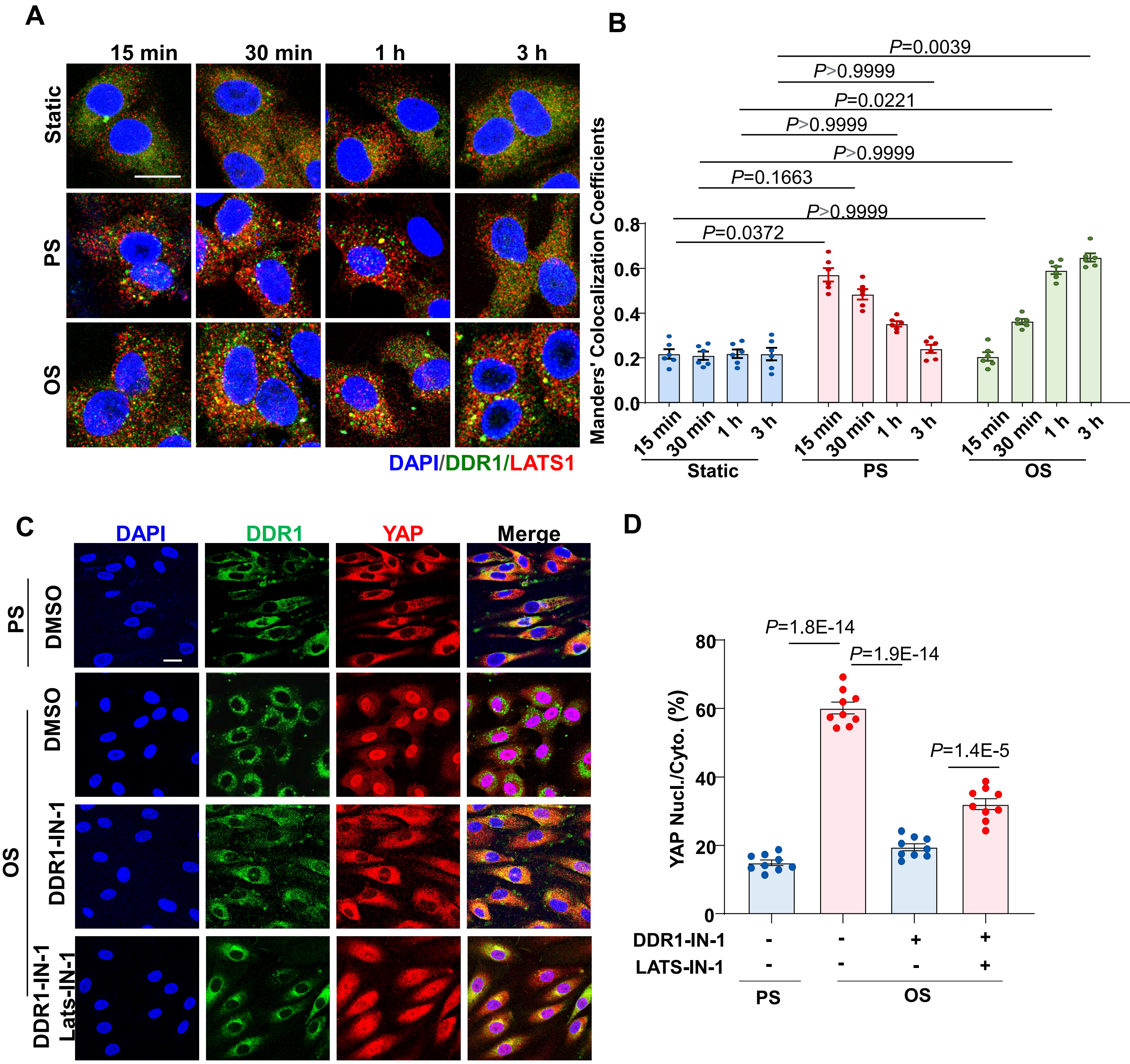


**Figure S13. DDR1 can interact with LATS1 to regulate YAP nuclear translocation partially.** (A) Representative Immunofluorescence staining of DDR1 and LATS1 in HUVECs subjected to PS or OS for four time periods (15 min, 30 min, 1 h and 3 h). (B) Co-location analysis of DDR1 and LATS1. Manders' colocalization coefficients was calculated by ImageJ Fiji software (Analyze-colocalization-coloc2), representing the proportion of LATS1 (co-located with DDR1) to the total LATS1. n=6 images from 3 biological replicates. Data were expressed as the means ± SEM and analyzed by Kruskal-Wallis test with Dunn's test. (C,D) Immunofluorescence and quantification of YAP localization in HUVECs. The cells were subjected to PS or OS for 24 hours, and treated wit hinhibitors or DMSO. n= 9 images from 3 biological replicates. 10~15 cells per image. Data were expressed as the means ± SEM and analyzed by two-way ANOVA followed by Tukey's multiply test. Scale bars, 20 μm.

## SUPPLEMENTARY TABLES

**Table S1: 64 proteins selected with a fold-change (OS/PS) > 1.25 or < 0.75 in Co-IP mass spectrometry.**

| Gene Name | Abundances (Normalized): OS | Abundances (Normalized): PS | Abundances (Normalized): Static | Fold Change (OS/PS) |
|---|---|---|---|---|
| HSPB1 | 414789.2388 | Not detected | Not detected | ∞ |
| PIK3CB | 226643.986 | Not detected | 71159.2542 | ∞ |
| TPI | 129236.2009 | Not detected | 152846.5244 | ∞ |
| VCP | 40654.493 | Not detected | 62338.88264 | ∞ |
| VIM | 49547078.08 | 19489685.16 | 127189802.8 | 2.542220547 |
| CKB | 737320.8495 | 307087.6719 | 326194.486 | 2.401010907 |
| FUS | 338556.8745 | 145159.7656 | 149319.4473 | 2.332305188 |
| PIN1 | 143548.7231 | 63813.28125 | 97453.85217 | 2.249511705 |
| SFPQ | 385847.6719 | 210576.7344 | 135991.2527 | 1.832337618 |
| APOB | 241790.6156 | 136502.4551 | 200255.2553 | 1.771327963 |
| YWHAE | 381842.5701 | 219940.7813 | 57298.45542 | 1.736115367 |
| FLNB | 558885.0254 | 332616.5703 | 161435.1003 | 1.680268139 |
| TXNDC5 | 2973484.988 | 1826692.203 | 2504556.567 | 1.627797493 |
| LTF | 633702.5845 | 392442.0234 | 512052.0581 | 1.614767397 |
| PDIA6 | 2393447.301 | 1485056.578 | 1328970.061 | 1.61168762 |
| SERPINB3 | 343245.1051 | 224773.1875 | 170386.8543 | 1.527073175 |
| MBNL1 | 158607.7221 | 104213.3516 | 124500.33 | 1.521952031 |
| DDX5 | 2623081.859 | 1759252.063 | 7904626.391 | 1.491021051 |
| NONO | 885605.9419 | 601472.6875 | 974922.5387 | 1.472395938 |
| ALDOA | 223297.2517 | 151961.4531 | 137943.8183 | 1.469433512 |
| LDHB | 956874.0026 | 662269.3125 | 559929.2751 | 1.444841222 |
| EEF1G | 658101.9341 | 473052.75 | 610593.9586 | 1.391180865 |
| LYZ | 310555.5012 | 227400.4063 | 214195.2739 | 1.365676985 |
| RRBP1 | 184133.9759 | 138652.0469 | 186634.605 | 1.328029265 |
| ANGPT2 | 491283.0894 | 371794.375 | 483650.0464 | 1.321383868 |
| GNAI1 | 305754.1259 | 234926.125 | 217983.4941 | 1.301490526 |
| PHB1 | 207892.6303 | 162256.2813 | 171568.332 | 1.281260909 |
| PCNA | 35264.14275 | 27803.61523 | Not detected | 1.268329404 |
| TUFM | 304427.1644 | 243535.5078 | 176575.4793 | 1.250031945 |
| S100A8 | 500146.8482 | 683905.0625 | 273734.7413 | 0.731310346 |
| PHB2 | 600543.7433 | 821629.8125 | 544927.6193 | 0.730917664 |
| TUBB6 | 223063.0595 | 307143.4375 | 239724.5662 | 0.726250449 |
| DSC1 | 841909.9922 | 1191202.516 | 1110858.381 | 0.706773182 |
| RPN1 | 297375.6361 | 424372.625 | 206574.1289 | 0.700741798 |
| FSCN1 | 356627.5862 | 510809.5 | | 0.698161616 |
| A2M | 4455419.437 | 6428015.553 | 13474984.94 | 0.69312518 |
| CCT2 | 83675.17322 | 121838.6797 | 95556.61598 | 0.68677019 |

| | | | | |
|---|---|---|---|---|
| FLNA | 517993.8032 | 765010.0781 | 150108.7947 | 0.677107162 |
| FLG2 | 628103.6432 | 939397.0625 | 706090.3212 | 0.668624236 |
| CRIP2 | 195119.3378 | 293467.7813 | 150047.159 | 0.664874819 |
| LRRC59 | 94290.24725 | 142262.3438 | 88410.98399 | 0.662791325 |
| PXDN | 205745.4913 | 310519.1875 | 123220.499 | 0.662585436 |
| MIF | 305178.9154 | 462720.7969 | 222825.7556 | 0.659531444 |
| TUBB2C | 2933402.05 | 4458585.703 | 1668500.896 | 0.657922096 |
| NCL | 277883.2042 | 423537.3125 | 276165.7562 | 0.656100882 |
| GSR | 534675.7329 | 821973.8125 | 494052.2251 | 0.650477819 |
| SLC25A6 | 94502.69825 | 145730.6406 | 146630.8039 | 0.648475145 |
| RPS4X | 7967927.839 | 12363208.44 | 13703819.56 | 0.644487058 |
| SET | 51169.82559 | 82365.32813 | 42593.30066 | 0.621254437 |
| RAN | 782599.1111 | 1260666.75 | 746915.9318 | 0.620781909 |
| LCN1 | 146154.2419 | 243398.125 | 140887.416 | 0.600473984 |
| IBA57 | 106392.6437 | 182819.1094 | 183729.8119 | 0.581955814 |
| TUBA1C | 4782642.911 | 8434845.668 | 4590357.923 | 0.567010127 |
| RACK1 | 346625.661 | 624345.4063 | 151372.8418 | 0.555182528 |
| PTMAP7 | 306651.9555 | 557063.7813 | 522424.1056 | 0.550479076 |
| DDX3X | 146476.5173 | 277693.8125 | 363848.0347 | 0.527474905 |
| DSG1 | 941016.4541 | 1937544.859 | 978878.5812 | 0.485674667 |
| ERP70 | 105887.4437 | 225026.5313 | 59177.73485 | 0.470555375 |
| CAPZA2 | 74534.69717 | 160005.4844 | 153121.4909 | 0.46582589 |
| DDX6 | 131358.6917 | 290641.5313 | 213603.618 | 0.451961188 |
| CLTC | 85728.69801 | 219999.25 | 80074.51422 | 0.389677228 |
| C1R | 55129.98609 | 147060.9102 | 261390.9066 | 0.374878586 |
| APOH | 40771.90243 | 139749.0352 | 171549.7699 | 0.291750869 |
| GAPDH | 1226515.331 | 4847751.625 | 1103041.324 | 0.253007049 |

**Table S2: 17 proteins predicted to be physically interacted with DDR1 by PRISM**

| Gene Name | Abundances (Normalized): OS | Abundances (Normalized): PS | Fold Change (OS/PS) | Identifier of protein | Binding energy predicted by PRISM |
|---|---|---|---|---|---|
| PIK3CB | 226643.986 | Not detected | ∞ | AF-P42338-F1 | -16.58 |
| PIN1 | 143548.7231 | 63813.28125 | 2.249511705 | AF-Q13526-F1 | -18.17 |
| YWHAE | 381842.5701 | 219940.7813 | 1.736115366 | 2BR9 | -17.63 |
| TXNDC5 | 2973484.988 | 1826692.203 | 1.627797493 | 3WGD | -22.92 |
| LTF | 633702.5845 | 392442.0234 | 1.614767397 | AF-P02788-F1 | -2.01 |
| PDIA6 | 2393447.301 | 1485056.578 | 1.611687619 | AF-Q15084-F1 | -8.54 |
| ANGPT 2 | 491283.0894 | 371794.375 | 1.321383868 | AF-O15123-F1 | -56.2 |
| YWHAQ | Not detected | 136783.6563 | 0 | AF-P27348-F1 | -42.23 |
| CLTC | 85728.69801 | 219999.25 | 0.389677228 | AF-Q00610-F1 | -14.02 |

| | | | | | |
|---|---|---|---|---|---|
| RACK1 | 346625.661 | 624345.4063 | 0.555182528 | 4AOW | -13.72 |
| LCN1 | 146154.2419 | 243398.125 | 0.600473984 | AF-P31025-F1 | -35.63 |
| SET | 51169.82559 | 82365.32813 | 0.621254437 | AF-Q01105-F1 | -22.81 |
| CRIP2 | 195119.3378 | 293467.7813 | 0.664874818 | AF-P52943-F1 | -3.58 |
| CCT2 | 83675.17322 | 121838.6797 | 0.68677019 | AF-P78371-F1 | -6.91 |
| FSCN1 | 356627.5862 | 510809.5 | 0.698161616 | AF-Q16658-F1 | -24.28 |
| RPN1 | 297375.6361 | 424372.625 | 0.700741798 | AF-P04843-F1 | -14.4 |
| PHB2 | 600543.7433 | 821629.8125 | 0.730917664 | AF-Q99623-F1 | -35.08 |

**Table S3: Primers for qPCR**

| Primer name | Forward (5'-3') | Reverse (5'-3') |
|---|---|---|
| MCP1 | CAGCCAGATGCAATCAATGCC | TGGAATCCTGAACCCACTTCT |
| E-selectin | TGGTGAGGTGTGCTCATTCC | TGATCTTTCCCGGAACTGCC |
| KLF4 | ACCAGGCACTACCGTAAACACA | GGTCCGACCTGGAAAATGCT |
| KLF2 | CTTCTCTCCCACCGGGTCTA | TAGCCCAAAAATGCCCACCT |
| CTGF | AGGAGTGGGTGTGTGACGA | CCAGGCAGTTGGCTCTAATC |
| CYR61 | TGAAGCGGCTCCCTGTTTT | CGGGTTTCTTTCACAAGGCG |
| ANKRD1 | AGTAGAGGAACTGGTCACTGG | TGGGCTAGAAGTGTCTTCAGAT |
| DDR1 | CCGACTGGTTCGCTTCTACC | CGGTGTAAGACAGGAGTCCATC |
| YWHAE | GATTCGGGAATATCGGCAAATGG | GCTGGAATGAGGTGTTTGTCC |
| PIEZO1 | GGCTGTCACTGAGAGGATGTTCA | AGCCACAGCGGATCTGGTA |
| GAPDH | AAGGTGAAGGTCGGAGTCAA | AATGAAGGGGTCATTGATGG |
| mGapdh | AGGTCGGTGTGAACGGATTTG | TGTAGACCATGTAGTTGAGGTCA |
| mDdr1 | GCTCTCCAATCCGGCCTAC | CGGGCTCCATATAGTCCCCA |
| mDgat1 | TTCCGCCTCTGGGCATT | AGAATCGGCCCACAATCCA |
| mGpam | CACACGAGCAGGAAAGATGA | GGACTGCATAGATGCTGCAA |
| mScd1 | TTCTTGCGATACACTCTGGTGC | CGGGATTGAATGTTCTTGTCGT |
| mPparg | TCAGCTCTGTGGACCTCTCC | ACCCTTGCATCCTTCACAAG |
| mCd36 | ACAGACGCAGCCTCCTTT | GTCGATTTCAGATCCGAACA |
| mFatp1 | CAGTGCCACCAACAAGAAGA | CAGCTCGTCCATCACTAGCA |
| mFabp4 | ACACCGAGATTTCCTTCAAACTG | CCATCTAGGGTTATGATGCTCTTCA |
| mFatp4 | ACTGTTCTCCAAGCTAGTGCT | GATGAAGACCCGGATGAAACG |
| mMacd | TTACCGAAGAGTTGGCGTATGG | TGCGGAGGGCTCTGTCAC |
| mLcad | CTCCCTGCGCGTCCTGAG | AAAATGTCATGCTCCGAGGAAAAG |
| mVlcad | GCCCAGACACACAACCTTTG | CCGAGCCGACTGCATCTC |
| mUcp1 | GTGAACCCGACAACTTCCGAA | TGCCAGGCAAGCTGAAACTC |
| mPrdm16 | TGCTGACGGATACAGAGGTGT | CCACGCAGAACTTCTCGCTAC |
| mPgc1a | TGCGGGATGATGGAGACA | TCGTTCGACCTGCGTAAAG |

**Table S4: Primers for plasmid construction**

| Plasmids | Primer | sequence(5'to3') |
|---|---|---|
| DDR1 Point mutants | F-W53A | ATCTCTGCTTCCAGCTCCGCGTCAGATTCCA |
| | R-W53A | GCGGAGCTGGAAGCAGAGATGTCACTGTCTG |

| | | |
|---|---|---|
| | F-K674A | GATTTCCTGAAAGAGGTGGCGATCATGTCG |
| | R-K674A | GCCACCTCTTTCAGGAAATCATTCCTGGC |
| | F-S677A | GAAAGAGGTGAAGATCATGGCGAGGCTCAAGG |
| | R-S677A | GCCATGATCTTCACCTCTTTCAGGAAATC |
| | F-C287A | CAGGTCCACGCTAACAACATGCACACG |
| | R-C287A | AGCGTGGACCTGCATAGCCTGGAAGGC |
| DDR1(1-669)-EGFP | F-DDR1-669 | CTTGGTACCGAGCTCATGGGACCAGAGGCCCTGT |
| | R-DDR1-669 | ACCAGAGCCGAAATCATTCCTGGCATTCTTGGTGG |
| | F-EGFP | TTCCTGGGCTCTGGTATGGTGAGCAAGG |
| | R-EGFP | GTGCTGGATATCTGCATTACTTGTACAGCTCGTCCATGCC |
| DDR1(1-693)-EGFP | F-DDR1-693 | CTTGGTACCGAGCTCATGGGACCAGAGGCCCTGT |
| | R-DDR1-693 | ACCAGAGCCCTGCACACACACGCCCAG |
| | F-EGFP | TGTGCAGGGCTCTGGTATGGTGAGCAAGG |
| | R-EGFP | GTGCTGGATATCTGCATTACTTGTACAGCTCGTCCATGCC |
| YWHAE-Cherry | F-YWHAE | CTTGGTACCGAGCTCATGGATGATCGAGAGGATCTGGTGTAC |
| | R-YWHAE | ACCAGAGCCCTGATTTTCGTCTTCCACGTCCTGC |
| | F-Cherry | AAATCAGGGCTCTGGTATGGAGGAGGACAA |
| | R-Cherry | GTGCTGGATATCTGCATTATCCGGATC |
| YWHAE-Y214A | F-Y214A | ACGCTGAGTGAAGAAAGCGCTAAGGACTCTA |
| | R-Y214A | GCGCTTTCTTCACTCAGCGTATCCAGTTC |
| DDR2-EGFP | F-DDR2 | CTTGGTACCGAGCTCATGATCCTGATTCCCAGAATGCTC |
| | R-DDR2 | ACCAGAGCCCTCGTCGCCTTGTTGAAGGAG |
| | F-EGFP | GCGACGAGGGCTCTGGTATGGTGAGCAAGG |
| | R-EGFP | GTGCTGGATATCTGCATTACTTGTACAGCTCGTCCATGCC |
| VE-Cadherin-Cherry | F-VE-Cadherin | CTTGGTACCGAGCTCATGCAGAGGCTCATGATGCTCC |
| | R-VE-Cadherin | ACCAGAGCCATACAGCAGCTCCTCCCGG |
| | F-Cherry | TGCTGTATGGCTCTGGTATGGAGGAGGACAACATGGCC |
| | R-Cherry | GTGCTGGATATCTGCATTATCCGGATCCACCACCGGTAC |
| Opto-YWHAE | F-opto | CTTGGTACCGAGCTCATGAAGATGGACAAAAAGACTATAGTTTGGTTTAGAAG |
| | R-opto | ACCAGAGCCTCCGGATCCACCA |
| | F-YWHAE | ATCCGGAGGCTCTGGTATGGATGATCGAGAGGATCTGGTGT |
| | R-YWHAE | GTGCTGGATATCTGCATTACTGATTTTCGTCTTCCACGTCCTG |
| reDS-DDR1 | F-DS | GGTACCGAGCTCATGATCCTGATTC |
| | R-DS | CCAGACACAGCCGTAAAGCTC |
| | F-2 | ACGGCTGTGTCTGGAGGGATGGACTCCTGTCTTACA |
| | R-2 | GTGCTGGATATCTGCATTACTTGTACAGCTCGTCCATGCC |

| | | |
|---|---|---|
| reDSlike-DDR1 | F-DS | CTTGGTACCGAGCTCATGGGACCAGAGGCCCTGT |
| | R-DS | CCAGAGGCAGCCATAGAGCTC |
| | F-DSlike | TGGCTGCCTCTGGCTAGATGGCTTGGTGTCTTACAAT |
| | R-DSlike | ATCTGATTGGAAGGTGATCTCACTGAAC |
| | F-3 | CCTTCCAATCAGATGTGGTGAACAATTCCTCTCCGG |
| | R-3 | GTGCTGGATATCTGCATTACTTGTACAGCTCGTCCATGCC |
| DDR1-locked (D189C and F364C) | F-C1 | CTTGGTACCGAGCTCATGGGACCAGAGGCCCTGT |
| | R-C1 | ACACCTCCAGAGGCAGCCATAGAG |
| | F-C2 | TGCCTCTGGAGGTGTGGACTCCTGTCTTACACC |
| | R-C2 | GCAGGAGATTTCGCTGAAGAGTAACCAG |
| | F-3 | GCGAAATCTCCTGCATCTCTGATGTGGTGAAC |
| | R-3 | GTGCTGGATATCTGCATTACTTGTACAGCTCGTCCATGCC |
| mCherry-DDR1 (WT/locked) | F-mCherry | CTTGGTACCGAGCTCATGGTGAGCAAGGGCGAGGA |
| | R-mCherry | ACCAGAGCCCTTGTACAGCTCGTCCATGCC |
| | F-DDR1 | GTACAAGGGCTCTGGTATGGGACCAGAGGCCCTG |
| | R-DDR1 | GTGCTGGATATCTGCAttaCACCGTGTTGAGTGCATCCTC |

**Table S5: siRNAs specific for DDR1 and YWHAE**

| siRNA name | sense (5'-3') |
|---|---|
| si-DDR1 | GAAUGUCGCUUCCGGCGUGUU |
| si-YWHAE | UGAAAAAGCCUCUAUGUAGUC |
| si-Piezo1 | GCAAGUUCGUGCGCGGAUU |

**Table S6: Primers for mice genotyping**

| Primer name | sequence(5'to3') |
|---|---|
| F-DDR1 flox | TTAGGTAGGACCTGAAAGGACAGG |
| R-DDR1 flox | GAGTTCTGGATAGCCTGAAGGTTT |
| F-Cdh5-Cre | CCGGTCGATGCAACGAGTGATGAGG |
| R-Cdh5-Cre | GCCTCCAGCTTGCATGATCTCCGG |

**Legends of supplementary videos 1 and 2**

**Supplementary video 1. Three-dimensional reconstruction of DDR1 droplets in HUVECs under the static conditions.** HUVECs were infected with DDR1-EGFP recombinant adenovirus and seeded on gelatin-coated microfluidic chamber.

**Supplementary video 2. Three-dimensional reconstruction of DDR1 droplets in HUVECs subjected to laminar flow for 2 minutes.** HUVECs were infected with DDR1-EGFP recombinant adenovirus, seeded on microfluidic chamber and then subjected to laminar flow for 2 minutes.
