# Supplementary material for: Endothelial discoidin domain receptor 1 senses flow to modulate YAP activation
Source: Nat Commun. 2023 Oct 13;14:6457. doi: 10.1038/s41467-023-42341-z (PMC10576099; doi:10.1038/s41467-023-42341-z)
Supplement: Supplementary file 3 — Description of Additional Supplementary Files [file 41467_2023_42341_MOESM3_ESM.pdf]

Description of Additional Supplementary Files

File name: Supplementary Movie 1
Description: Three-dimensional reconstruction of DDR1 droplets in HUVECs under the static conditions. HUVECs were infected with DDR1-EGFP recombinant adenovirus and seeded on gelatin-coated microfluidic chamber.

File name: Supplementary Movie 2
Description: Three-dimensional reconstruction of DDR1 droplets in HUVECs subjected to laminar flow for 2 minutes. HUVECs were infected with DDR1-EGFP recombinant adenovirus, seeded on microfluidic chamber and then subjected to laminar flow for 2 minutes.
