# Supplementary material for: Endothelial discoidin domain receptor 1 senses flow to modulate YAP activation
Source: Nat Commun. 2023 Oct 13;14:6457. doi: 10.1038/s41467-023-42341-z (PMC10576099; doi:10.1038/s41467-023-42341-z)
Supplement: Supplementary file 6 — Reporting Summary [file 41467_2023_42341_MOESM6_ESM.pdf]

nature portfolio

Corresponding author(s): Jing Zhou
Last updated by author(s): Sep 28, 2023

Reporting Summary

Nature Portfolio wishes to improve the reproducibility of the work that we publish. This form provides structure for consistency and transparency in reporting. For further information on Nature Portfolio policies, see our Editorial Policies and the Editorial Policy Checklist.

Statistics

For all statistical analyses, confirm that the following items are present in the figure legend, table legend, main text, or Methods section.

n/a	Confirmed	
☐	☒	The exact sample size (*n*) for each experimental group/condition, given as a discrete number and unit of measurement
☐	☒	A statement on whether measurements were taken from distinct samples or whether the same sample was measured repeatedly
☐	☒	The statistical test(s) used AND whether they are one- or two-sided<br>*Only common tests should be described solely by name; describe more complex techniques in the Methods section.*
☐	☒	A description of all covariates tested
☐	☒	A description of any assumptions or corrections, such as tests of normality and adjustment for multiple comparisons
☐	☒	A full description of the statistical parameters including central tendency (e.g. means) or other basic estimates (e.g. regression coefficient) AND variation (e.g. standard deviation) or associated estimates of uncertainty (e.g. confidence intervals)
☐	☒	For null hypothesis testing, the test statistic (e.g. *F*, *t*, *r*) with confidence intervals, effect sizes, degrees of freedom and *P* value noted<br>*Give P values as exact values whenever suitable.*
☒	☐	For Bayesian analysis, information on the choice of priors and Markov chain Monte Carlo settings
☐	☒	For hierarchical and complex designs, identification of the appropriate level for tests and full reporting of outcomes
☐	☒	Estimates of effect sizes (e.g. Cohen's *d*, Pearson's *r*), indicating how they were calculated

Our web collection on statistics for biologists contains articles on many of the points above.

Software and code

Policy information about availability of computer code

Data collection	LAS X microcope software, UniProt database
Data analysis	GraphPad Prism version 8.0.1, ImageJ, Coloc 2 plugin of Fiji, Microsoft Excel

For manuscripts utilizing custom algorithms or software that are central to the research but not yet described in published literature, software must be made available to editors and reviewers. We strongly encourage code deposition in a community repository (e.g. GitHub). See the Nature Portfolio guidelines for submitting code & software for further information.

Data

Policy information about availability of data

All manuscripts must include a data availability statement. This statement should provide the following information, where applicable:

- Accession codes, unique identifiers, or web links for publicly available datasets
- A description of any restrictions on data availability
- For clinical datasets or third party data, please ensure that the statement adheres to our policy

Protein structure files downloaded from UniProt database (https://www.uniprot.org/) are listed as follows: DDR1 (6BRJ), PIK3CB (AF-P42338-F1), PIN1 (AF-Q13526-F1), YWHAE (2BR9), TXNDC5 (3WGD), LTF (AF-P02788-F1), PDIA6 (AF-Q15084-F1), ANGPT2 (AF-O15123-F1), YWHAQ (AF-P27348-F1), CLTC (AF-Q00610-F1), RACK1 (4AOW), LCN1 (AF-P31025-F1), SET (AF-Q01105-F1), CRIP2 (AF-P52943-F1), CCT2 (AF-P78371-F1), FSCN1 (AF-Q16658-F1), RPN1 (AF-P04843-F1), PHB2 (AF-Q99623-F1). Data supporting the findings of this work are available within the paper and its Supplementary Information files. The identifier of each protein structure we

used for protein-protein interaction prediction were listed in Table S2 in the Data Supplement. All primers are listed in the Tables in the Data Supplement. Source data are provided with this paper and uploaded in Figshare (Doi:10.6084/m9.figshare.24204111).

Research involving human participants, their data, or biological material

Policy information about studies with human participants or human data. See also policy information about sex, gender (identity/presentation), and sexual orientation and race, ethnicity and racism.

Reporting on sex and gender	The umbilical cords are obtained from healthy women after full-term deliveries. The gender of the fetus includes both males and females. During the application, endothelial cells from 3-5 umbilical cords are mixed together.
Reporting on race, ethnicity, or other socially relevant groupings	yellow race
Population characteristics	puerperas aged 20-35 with full-term deliveries.
Recruitment	Obstetrics and Gynecology Department of Peking University People's Hospital
Ethics oversight	Be approved by the Peking University People's Hospital Medical Ethics committee (2015PHB024)

Note that full information on the approval of the study protocol must also be provided in the manuscript.

Field-specific reporting

Please select the one below that is the best fit for your research. If you are not sure, read the appropriate sections before making your selection.

☒ Life sciences ☐ Behavioural & social sciences ☐ Ecological, evolutionary & environmental sciences

For a reference copy of the document with all sections, see nature.com/documents/nr-reporting-summary-flat.pdf

Life sciences study design

All studies must disclose on these points even when the disclosure is negative.

Sample size	We used G*Power 3.1.9.7 to conduct priori power calculations. For animal study, by using nonparametric test and setting power (1-β) to 0.8, we obtained the required sample size per group as 6. For immunofluorescence experiment involving a group of cells, by using ANOVA test and setting power (1-β) to 0.8, we obtained the required sample size per group as 43 cells. So we ensured that at least 43 cells are included in the images collected in 3 biological replicates. For single-molecule magnetic tweezers measurements, by using matched Wilcoxon matched-pairs signed rank test and setting power (1-β) to 0.8, we obtained the required sample size per group as 7. For single cell live imaging, we conducted 8 or 9 biological replicates considering the heterogeneity of cells and experimental costs comprehensively. For qPCR experiments, we chose a commonly used biological replicates (n=6). For western blots assay, we conducted 3 biological replicates, because there are results from different time points in the same batch of experiments that can be used as self control.
Data exclusions	no data were excluded.
Replication	Experiments were replicated every 1-3 weeks independently.
Randomization	All allocations were random in this study.
Blinding	In the data collection and analysis of animal studies, only one investigator knew the genotype of each mouse, another investigator who is responsible for data collection only knew the corresponding number of each mouse. For cellular immunofluorescence assay and magnetic tweezers experiments, one investigator was responsible for conducting cell or single-molecule experiments and assigning group numbers, while another investigator was responsible for data collection and analysis without knowing the specific group. For Western Blot and qPCR assay, blinding were not used because the data needs to be presented in a certain grouping order.

Reporting for specific materials, systems and methods

We require information from authors about some types of materials, experimental systems and methods used in many studies. Here, indicate whether each material, system or method listed is relevant to your study. If you are not sure if a list item applies to your research, read the appropriate section before selecting a response.

Materials & experimental systems

n/a	Involved in the study
☐	☒ Antibodies
☐	☒ Eukaryotic cell lines
☒	☐ Palaeontology and archaeology
☐	☒ Animals and other organisms
☒	☐ Clinical data
☒	☐ Dual use research of concern
☒	☐ Plants

Methods

n/a	Involved in the study
☒	☐ ChIP-seq
☒	☐ Flow cytometry
☒	☐ MRI-based neuroimaging

Antibodies

Antibodies used	Primary antibodies against pDDR1-Y792(11994S; CST; 1:1000), DDR1 (10536-1-AP; Proteintech; 1:1000), YAP1 (13584-1-AP; Proteintech; 1:1000), pYAP1-S127 (AP0489; ABclonal; 1:1000), YWHAE (11648-2-AP; Proteintech; 1:1000) VE-cadherin (bs-4310R; Bioss; 1:1000) and GAPDH (BE0024; Easybio; 1:4000) were used for Western blotting. Rhodamine Phalloidin (RM02835; ABclonal; 1:200), Primary antibodies against YAP1 (66900-1-Ig; Proteintech; 1:200), DDR1 (10536-1-AP; Proteintech; 1:200), VE-Cadherin (sc-9989; Santa Cruz; 1:100), E-selectin (MA1-22165; Invitrogen; 1:200), ICAM1 (sc-7891; Santa Cruz; 1:100), VCAM1 (sc-13160; Santa Cruz; 1:100), Caveolin-1 (66067-1-Ig; Proteintech; 1:200), Rab 5A (sc-166600; Santa Cruz; 1:100), PE-conjugated CD63 (BC-353003; BaiCheng Technology; 1:200) and LATS1 (3477S; CST; 1:200) were used for immunofluorescence staining. DDR1 antibody (200 μg/ml; sc-390268; Santa Cruz) were used for bead pulling/magnetic tweezer system.
Validation	The validation of all primary antibodies for the species and application were described on the manufacturer's website.

Eukaryotic cell lines

Policy information about cell lines and Sex and Gender in Research

Cell line source(s)	HUVECs were isolated from umbilical cords from healthy patients after full-term deliveries. De-identified umbilical cords were obtained with the agreement of the patients and approved by the Peking University People's Hospital Medical Ethics Committee (2015PHB024).EA.hy926 (CL-0272) and NIH3T3 cells (CL-0171) was obtained from Pricella Biotechnology (Wuhan, China).
Authentication	EA.hy926 and NIH3T3 cells were authenticated using STR (short tandem repeats) testing.
Mycoplasma contamination	All cell lines were tested negative for mycoplasma contamination.
Commonly misidentified lines (See ICLAC register)	No.

Animals and other research organisms

Policy information about studies involving animals; ARRIVE guidelines recommended for reporting animal research, and Sex and Gender in Research

Laboratory animals	Wild type mice (C57BL6J, 8-10 weeks old) were obtained from the Experimental Animal Center at Peking University Health Science Center (Beijing, China). Mice carrying the Ddr1 coding region flanked by loxP sites (Ddr1 flox/flox) and were generated by Cyagen Biosciences (China). Cdh5-CreERT2 mice were obtained from Taconic Biosciences (model number: 13073). All animals were on the C57BL6J background. 7-week old Cre/LoxP-based conditional knockout mice and control littermates were subjected to tamoxifen, and partial ligation of the carotid artery was performed at 8-week. Mice were sacrificed at week-9 or week-12.
Wild animals	Not involve wild animals.
Reporting on sex	Ddr1 flox/flox mice were maintained in a C57BL/6 background and crossed with vascular endothelial-cadherin Cre recombinase-positive mice (Cdh5-CreERT2), to generate the Ddr1flox/flox Cdh5-CreERT2+ (Ddr1iECKO) mice. Ddr1flox/flox Cdh5-CreERT2- (Ddr1WT) littermates were used as the controls. 4 male mice and 3 female mice were included in each group of partial carotid ligation.
Field-collected samples	Mice were housed under controlled conditions with 23±1 °C of ambient temperature and 60% of relative humidity. They were synchronized to a 12-hour light-dark cycles with free access to food and water.
Ethics oversight	All animal studies were performed in accordance with the guidelines of the Animal Care and Use Committee of Peking University and approved by the Ethics Committee of Peking University Health Science Center (LA2015017).

Note that full information on the approval of the study protocol must also be provided in the manuscript.
