# Supplementary material for: Endothelial discoidin domain receptor 1 senses flow to modulate YAP activation
Source: Nat Commun. 2023 Oct 13;14:6457. doi: 10.1038/s41467-023-42341-z (PMC10576099; doi:10.1038/s41467-023-42341-z)

Figure 2C

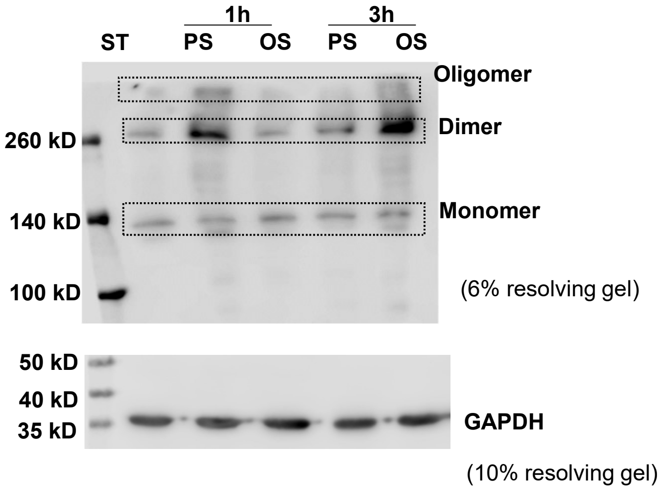

Figure 2E

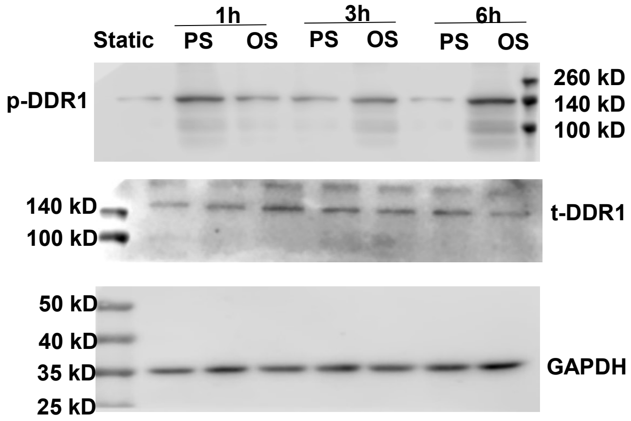

Figure 2E

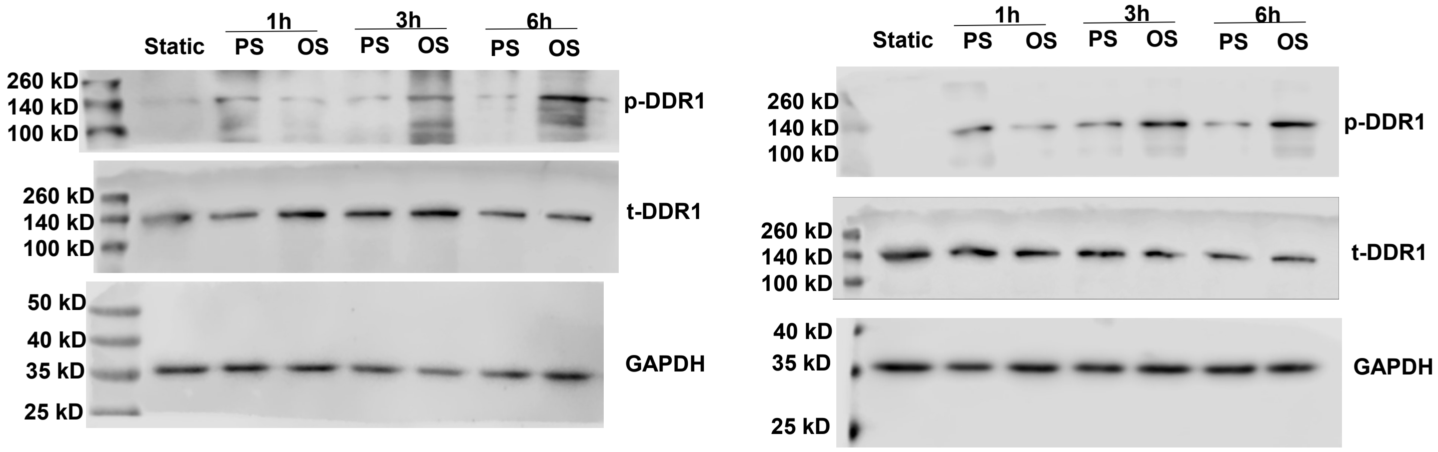

Figure 3K

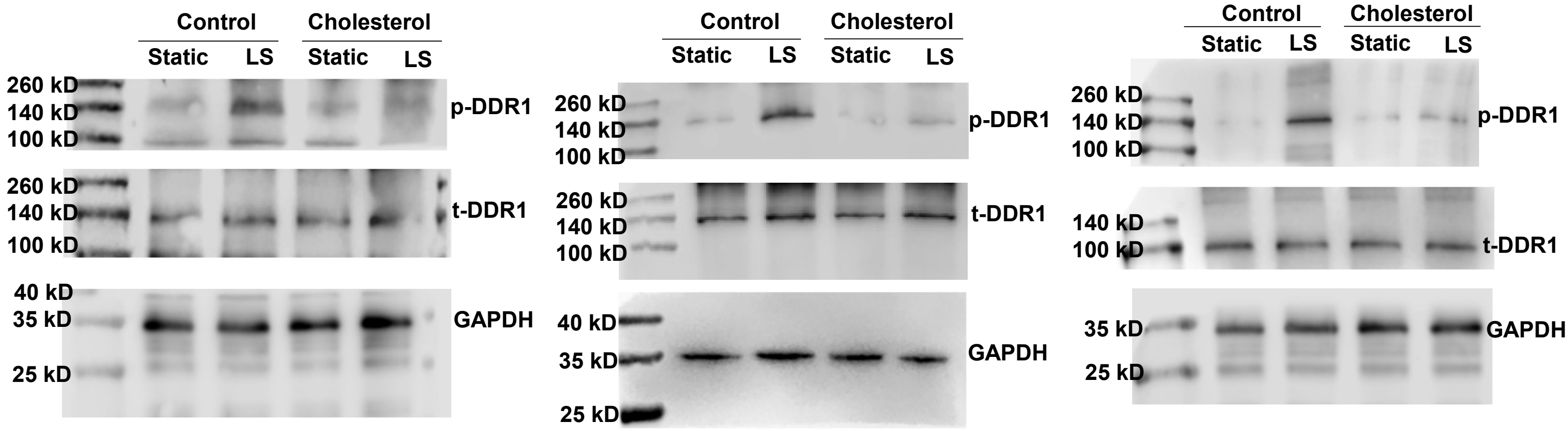

Figure 4L

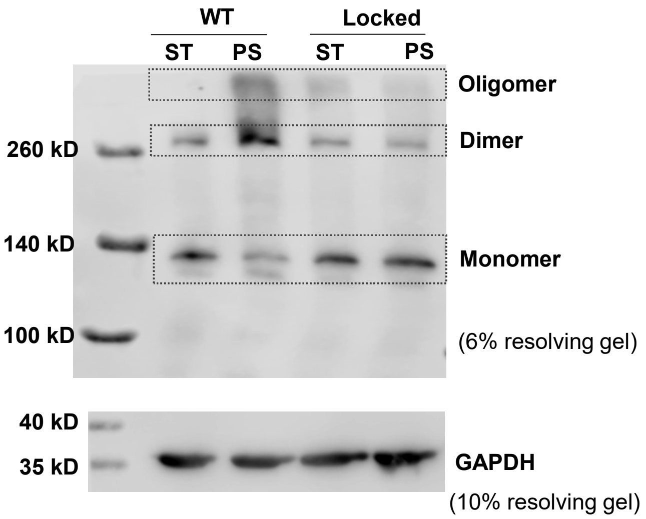

Figure 5B

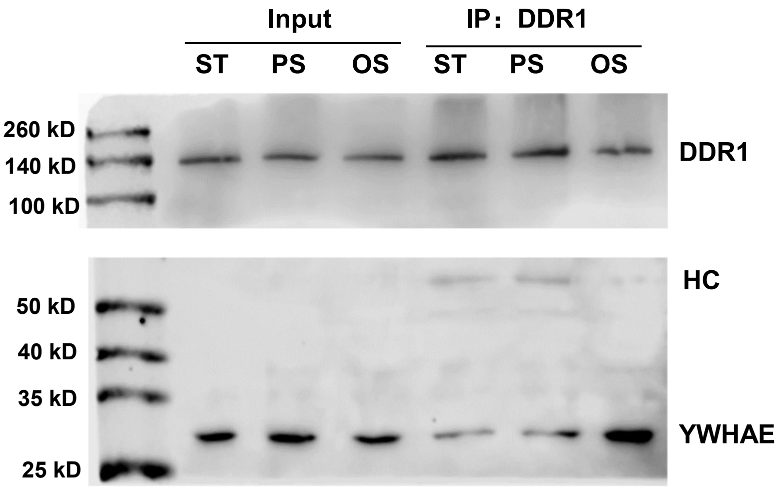

Figure 6D

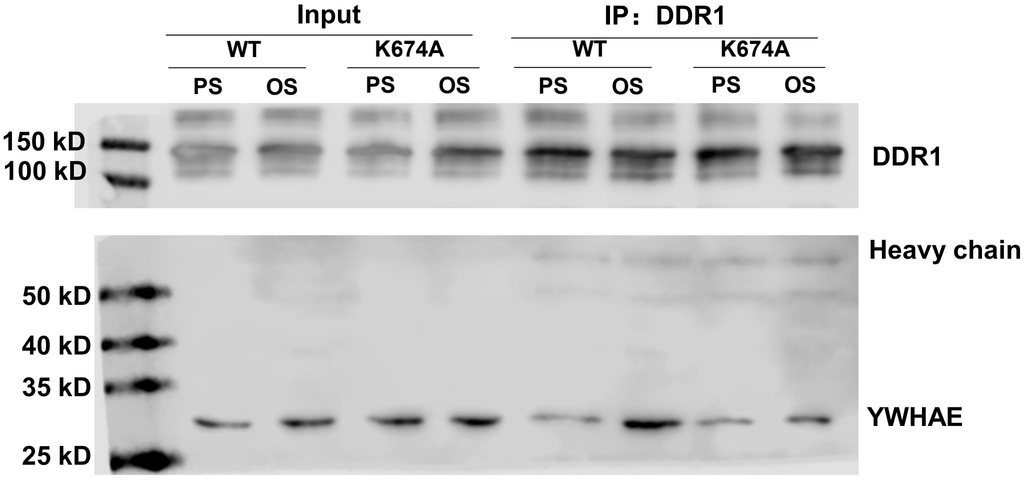

Figure 7B

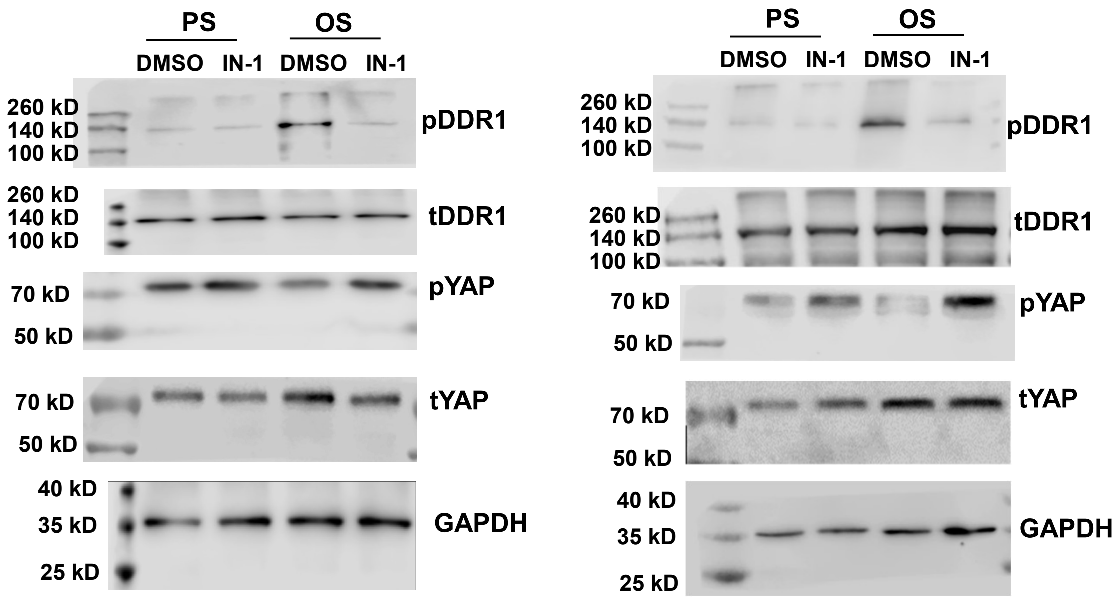

Figure 7F

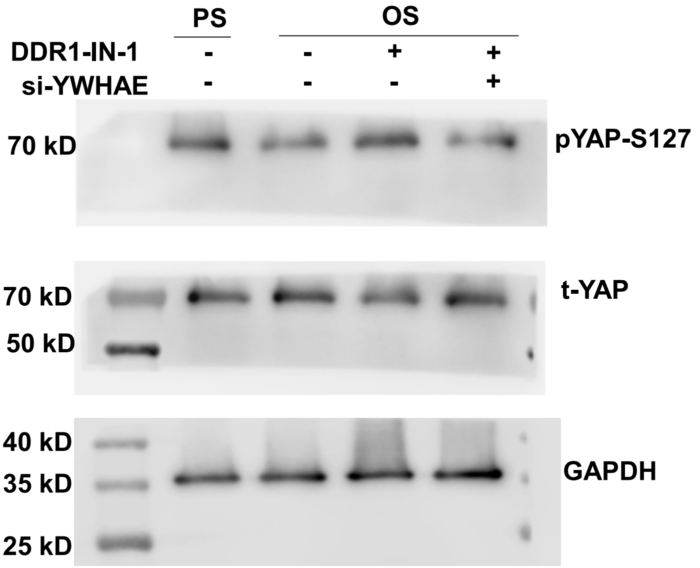

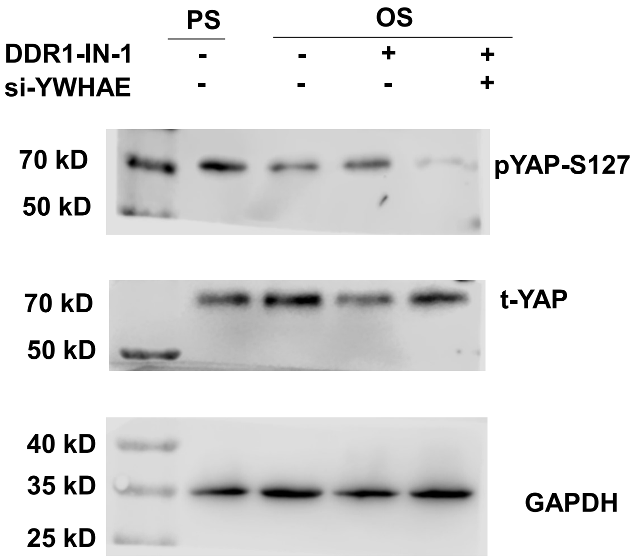

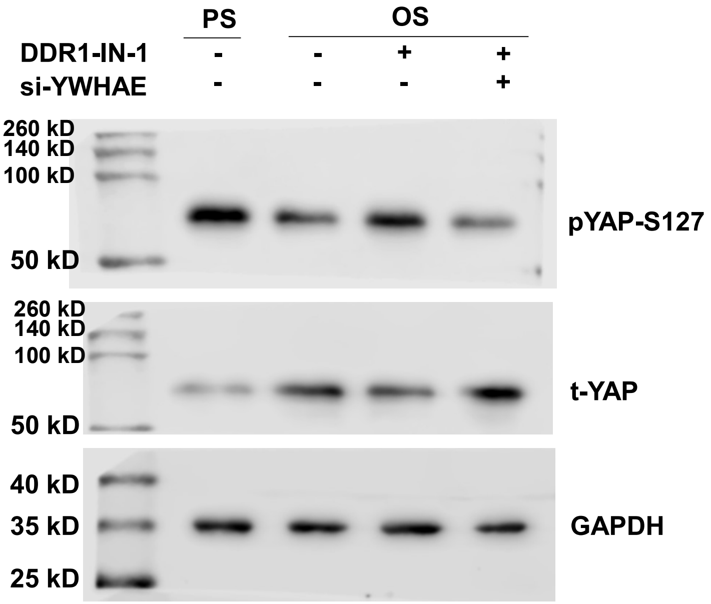

Figure S1B

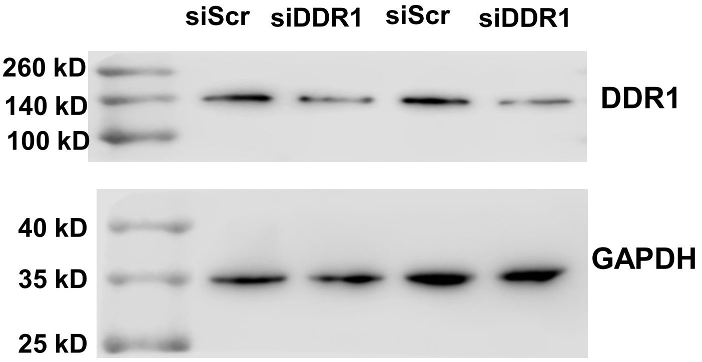

Figure S4A

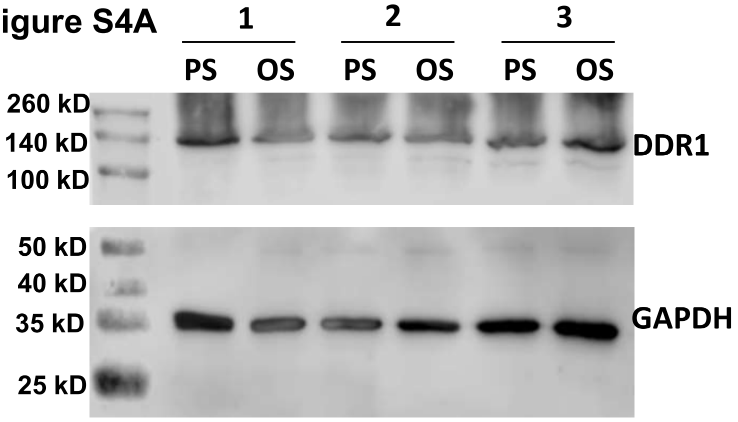

Figure S6E

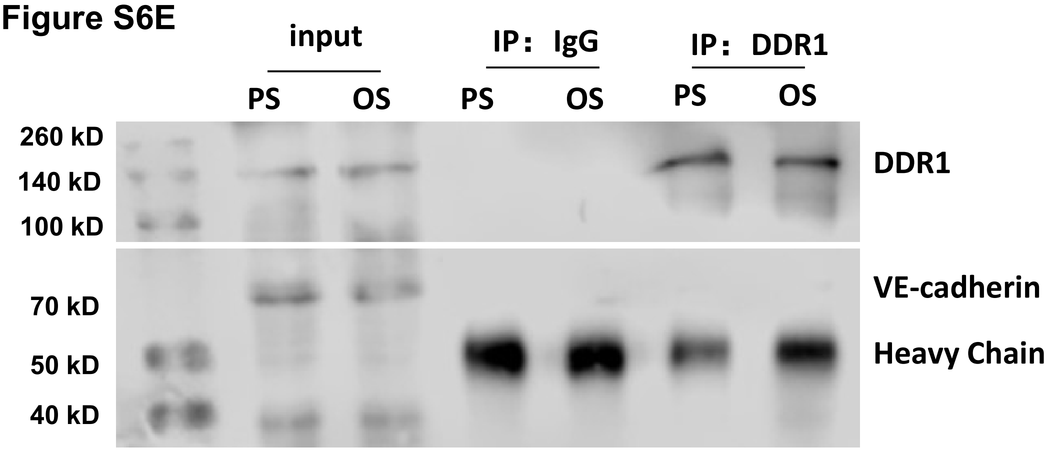

Supplement: Supplementary file 7 — Source Data [file 41467_2023_42341_MOESM7_ESM.zip › gels.pdf]
